# Supplementary material for: QuantumBind-RBFE: Accurate Relative Binding Free Energy Calculations Using Neural Network Potentials
Source: J Chem Inf Model. 2025 Apr 8;65(8):4081–9. doi: 10.1021/acs.jcim.5c00033 (PMC12131219; doi:10.1021/acs.jcim.5c00033)
Supplement: Supplementary file 1 [file ci5c00033_si_001.pdf]

Supporting Information

QuantumBind-RBFE: Accurate Relative Binding Free Energy Calculations Using Neural Network Potentials

Francesc Sabanés Zariquiey,[†] Stephen E. Farr,[†] Stefan Doerr,[‡] and Gianni De Fabritiis*,[¶,‖]

†*Acellera Labs, C Dr Trueta 183, 08005, Barcelona, Spain*

‡*Acellera Therapeutics, 38350 Fremont Blvd 203 Fremont CA, 94536 USA*

¶*Computational Science Laboratory, Universitat Pompeu Fabra, Barcelona Biomedical Research Park (PRBB), C Dr. Aiguader 88, 08003, Barcelona, Spain*

§*Acellera Therapeutics, 38350 Fremont Blvd 203 Fremont CA, 94536 USA*

‖*Institució Catalana de Recerca i Estudis Avançats (ICREA), Passeig Lluis Companys 23, 08010 Barcelona, Spain*

E-mail: g.defabritiis@acellera.com

Table S1: Alchemical schedule of the Solftplus Alchemical Potential for the two legs for the alchemical transformations. α values are in $(\text{kcal/mol})^{-1}$ and u_0 and w_0 are in kcal/mol

λ	λ_1	λ_2	α	u_0	w_0
0.00	0.00	0.00	0.10	110	0
0.05	0.00	0.10	0.10	110	0
0.10	0.00	0.20	0.10	110	0
0.15	0.00	0.30	0.10	110	0
0.20	0.00	0.40	0.10	110	0
0.25	0.00	0.50	0.10	110	0
0.30	0.10	0.50	0.10	110	0
0.35	0.20	0.50	0.10	110	0
0.40	0.30	0.50	0.10	110	0
0.45	0.40	0.50	0.10	110	0
0.50	0.50	0.50	0.10	110	0
0.55	0.50	0.50	0.10	110	0
0.60	0.40	0.50	0.10	110	0
0.65	0.30	0.50	0.10	110	0
0.70	0.20	0.50	0.10	110	0
0.75	0.10	0.50	0.10	110	0
0.80	0.00	0.40	0.10	110	0
0.85	0.00	0.30	0.10	110	0
0.90	0.00	0.20	0.10	110	0
0.95	0.00	0.10	0.10	110	0
1.00	0.00	0.00	0.10	110	0

0.1 ΔG values

Table S2: Comparison of the performance of different ligand force fields: GAFF2, OPLS4, and AceFF 1.0. Root Mean Square Error (RMSE) in kcal/mol and Kendall correlation (τ) for the 8 tested protein targets. 95% confidence intervals are shown with lower and upper bounds as subscripts and superscripts.

**Target**	**GAFF2**			**OPLS4 (FEP+)**[1]			**AceFF 1.0**			$N_{\mathbf{lig}}$
	RMSE	MAE	Kendall (τ)	RMSE	MAE	Kendall (τ)	RMSE	MAE	Kendall (τ)	
BACE	$1.07^{1.27}_{0.85}$	$0.87^{1.08}_{0.65}$	$0.33^{0.56}_{0.06}$	$\mathbf{0.87}^{1.08}_{0.67}$	$\mathbf{0.71}^{0.88}_{0.55}$	$0.46^{0.65}_{0.23}$	$0.99^{1.24}_{0.75}$	$0.80^{1.00}_{0.61}$	$\mathbf{0.48}^{0.67}_{0.26}$	36
CDK2	$1.03^{1.37}_{0.62}$	$0.79^{1.11}_{0.51}$	$0.45^{0.80}_{0.13}$	$\mathbf{0.92}^{1.17}_{0.60}$	$0.72^{1.00}_{0.45}$	$0.45^{0.76}_{0.07}$	$0.94^{1.36}_{0.46}$	$\mathbf{0.64}^{1.03}_{0.33}$	$\mathbf{0.68}^{0.88}_{0.41}$	16
JNK1	$1.03^{1.28}_{0.76}$	$0.83^{1.11}_{0.59}$	$0.54^{0.73}_{0.29}$	$\mathbf{0.72}^{0.90}_{0.52}$	$\mathbf{0.59}^{0.78}_{0.42}$	$\mathbf{0.68}^{0.85}_{0.47}$	$1.15^{1.50}_{0.80}$	$0.92^{1.20}_{0.65}$	$0.55^{0.73}_{0.35}$	21
MCL1	$1.64^{1.95}_{1.31}$	$1.32^{1.64}_{1.05}$	$0.44^{0.60}_{0.25}$	$\mathbf{0.84}^{1.07}_{0.63}$	$\mathbf{0.65}^{0.82}_{0.50}$	$\mathbf{0.58}^{0.71}_{0.42}$	$1.07^{1.23}_{0.90}$	$0.91^{1.10}_{0.73}$	$0.28^{0.49}_{0.09}$	42
P38	$0.99^{1.30}_{0.70}$	$0.75^{0.97}_{0.53}$	$0.64^{0.78}_{0.45}$	$\mathbf{0.75}^{0.94}_{0.57}$	$\mathbf{0.58}^{0.74}_{0.42}$	$0.55^{0.70}_{0.36}$	$0.89^{1.16}_{0.65}$	$0.67^{0.87}_{0.49}$	$\mathbf{0.72}^{0.82}_{0.58}$	34
THROMBIN	$0.82^{1.08}_{0.47}$	$0.62^{0.93}_{0.32}$	$0.55^{0.96}_{0.00}$	$\mathbf{0.57}^{0.77}_{0.32}$	$\mathbf{0.45}^{0.66}_{0.27}$	$\mathbf{0.60}^{0.92}_{0.19}$	$0.80^{1.12}_{0.49}$	$0.64^{0.93}_{0.38}$	$0.42^{0.84}_{-0.15}$	11
TYK2	$0.65^{0.82}_{0.44}$	$0.52^{0.71}_{0.35}$	$0.67^{0.84}_{0.42}$	$\mathbf{0.45}^{0.63}_{0.24}$	$\mathbf{0.34}^{0.50}_{0.21}$	$\mathbf{0.80}^{0.96}_{0.56}$	$0.74^{0.92}_{0.53}$	$0.59^{0.83}_{0.37}$	$\mathbf{0.80}^{0.96}_{0.56}$	16
ALL	$1.17^{1.30}_{1.03}$	$0.90^{1.01}_{0.79}$	$0.55^{0.62}_{0.48}$	$\mathbf{0.78}^{0.88}_{0.69}$	$\mathbf{0.61}^{0.68}_{0.53}$	$\mathbf{0.66}^{0.72}_{0.60}$	$0.99^{1.10}_{0.89}$	$0.79^{0.89}_{0.71}$	$0.59^{0.65}_{0.52}$	176

0.2 Evaluating the accuracy of calculations

We analyzed the percentage of predictions that meet absolute error thresholds below 1 kcal/-mol and 2 kcal/mol to evaluate how well AceFF 1.0 minimizes outliers in comparison to the other described methods. Table S3 presents the percentage of values for each method across different protein targets within these thresholds, providing insight into the models' accuracy. At the 2 kcal/mol threshold, AceFF 1.0 calculations demonstrate competitive accuracy across most targets, with fewer outliers indicated by higher percentages within this error range. For example, on targets like BACE and P38, AceFF 1.0 generally outperforms GAFF2, demonstrating AceFF's 1.0 capacity to maintain accuracy across diverse systems. Similar trends are observed for THROMBIN and TYK2, where AceFF 1.0 shows strong performance relative to the other methods. For predictions within at the 1 kcal/mol threshold, AceFF 1.0 remains competitive with OPLS4 and GAFF2. On CDK2, JNK1, and MCL1, AceFF 1.0 performs closely to OPLS4. Notably, on TYK2, AceFF achieves a higher percentage of values within the 1 kcal/mol range than either OPLS4 or GAFF2. Overall, while OPLS4 reaches the highest absolute accuracy on some individual targets, AceFF 1.0 provides robust, consistent performance across both error thresholds, achieving comparable or superior accuracy across

a range of protein targets.

Table S3: Percentage of predictions with a mean absolute error (MAE) below 1 kcal/mol and 2 kcal/mol for each target, comparing the performance of GAFF2, OPLS4 with FEP+, and AceFF 1.0, providing insight into each method2019s ability to minimize outliers and maintain accuracy across diverse protein systems.

**Target**	**GAFF2**		**OPLS4 (FEP+)**		**AceFF 1.0**	
	**% MAE < 1**	**% MAE < 2**	**% MAE < 1**	**% MAE < 2**	**% MAE < 1**	**% MAE < 2**
**BACE**	60	78	59	93	66	93
**CDK2**	44	76	52	96	81	88
**JNK1**	65	91	71	100	56	82
**MCL1**	52	73	61	90	49	82
**P38**	71	95	66	100	63	93
**PTP1B**	59	84	94	98	57	82
**THROMBIN**	56	81	63	94	63	94
**TYK2**	78	96	79	96	92	100

0.3 $\Delta\Delta G$ values

Table S4: Comparison of the performance of different ligand force fields: GAFF2, OPLS4 with FEP+[1], and AceFF 1.0. Root Mean Square Error (RMSE) in kcal/mol and Kendall correlation (τ) for the 8 tested protein targets. 95% confidence intervals are shown with lower and upper bounds as subscripts and superscripts.

**Target**	**GAFF2**			**OPLS4 (FEP+)**			**AceFF 1.0**			$N_{\mathbf{edge}}$
	RMSE	MAE	Kendall (τ)	RMSE	MAE	Kendall (τ)	RMSE	MAE	Kendall (τ)	
BACE	$1.48_{1.20}^{1.75}$	$1.16_{0.92}^{1.39}$	$0.29_{0.10}^{0.48}$	$\mathbf{1.02}_{0.85}^{1.19}$	$\mathbf{0.82}_{0.67}^{0.98}$	$0.37_{0.20}^{0.51}$	$1.32_{1.04}^{1.61}$	$1.04_{0.84}^{1.27}$	$\mathbf{0.46}_{0.31}^{0.58}$	58
CDK2	$1.50_{1.14}^{1.83}$	$1.21_{0.87}^{1.58}$	$0.20_{-0.09}^{0.48}$	$\mathbf{1.13}_{0.89}^{1.33}$	$0.97_{0.74}^{1.20}$	$0.32_{0.01}^{0.55}$	$1.32_{0.85}^{1.71}$	$\mathbf{0.88}_{0.52}^{1.28}$	$\mathbf{0.40}_{0.06}^{0.69}$	25
JNK1	$1.13_{0.83}^{1.43}$	$0.89_{0.69}^{1.15}$	$0.34_{0.07}^{0.59}$	$\mathbf{0.90}_{0.72}^{1.07}$	$\mathbf{0.72}_{0.53}^{0.89}$	$\mathbf{0.39}_{0.15}^{0.60}$	$1.09_{0.84}^{1.32}$	$0.86_{0.62}^{1.09}$	$0.37_{0.11}^{0.58}$	34
MCL1	$1.91_{1.52}^{2.30}$	$1.43_{1.15}^{1.74}$	$\mathbf{0.42}_{0.27}^{0.55}$	$\mathbf{1.17}_{1.01}^{1.34}$	$0.96_{0.80}^{1.11}$	$0.33_{0.17}^{0.46}$	$1.22_{1.01}^{1.42}$	$\mathbf{0.94}_{0.77}^{1.11}$	$0.41_{0.28}^{0.52}$	71
P38	$1.11_{0.80}^{1.39}$	$0.81_{0.63}^{1.02}$	$0.62_{0.50}^{0.73}$	$\mathbf{0.86}_{0.70}^{0.99}$	$\mathbf{0.67}_{0.52}^{0.82}$	$0.63_{0.51}^{0.73}$	$1.10_{0.91}^{1.28}$	$0.88_{0.72}^{1.05}$	$\mathbf{0.65}_{0.54}^{0.74}$	56
THROMBIN	$1.42_{0.97}^{1.85}$	$1.12_{0.72}^{1.55}$	$0.41_{0.12}^{0.69}$	$\mathbf{1.02}_{0.72}^{1.31}$	$\mathbf{0.86}_{0.61}^{1.15}$	$\mathbf{0.41}_{0.03}^{0.68}$	$1.36_{0.75}^{1.88}$	$1.00_{0.57}^{1.47}$	$0.18_{-0.20}^{0.52}$	16
TYK2	$0.88_{0.58}^{1.16}$	$0.66_{0.44}^{0.92}$	$0.53_{0.23}^{0.77}$	$0.85_{0.56}^{1.09}$	$0.66_{0.46}^{0.87}$	$\mathbf{0.65}_{0.44}^{0.81}$	$\mathbf{0.77}_{0.56}^{0.96}$	$\mathbf{0.57}_{0.37}^{0.76}$	$\mathbf{0.64}_{0.43}^{0.80}$	24
ALL	$1.46_{1.30}^{1.62}$	$1.09_{0.97}^{1.20}$	$0.42_{0.34}^{0.49}$	$\mathbf{1.01}_{0.94}^{1.09}$	$\mathbf{0.81}_{0.74}^{0.88}$	$0.43_{0.37}^{0.49}$	$1.22_{1.10}^{1.33}$	$0.94_{0.85}^{1.03}$	$\mathbf{0.46}_{0.40}^{0.51}$	284

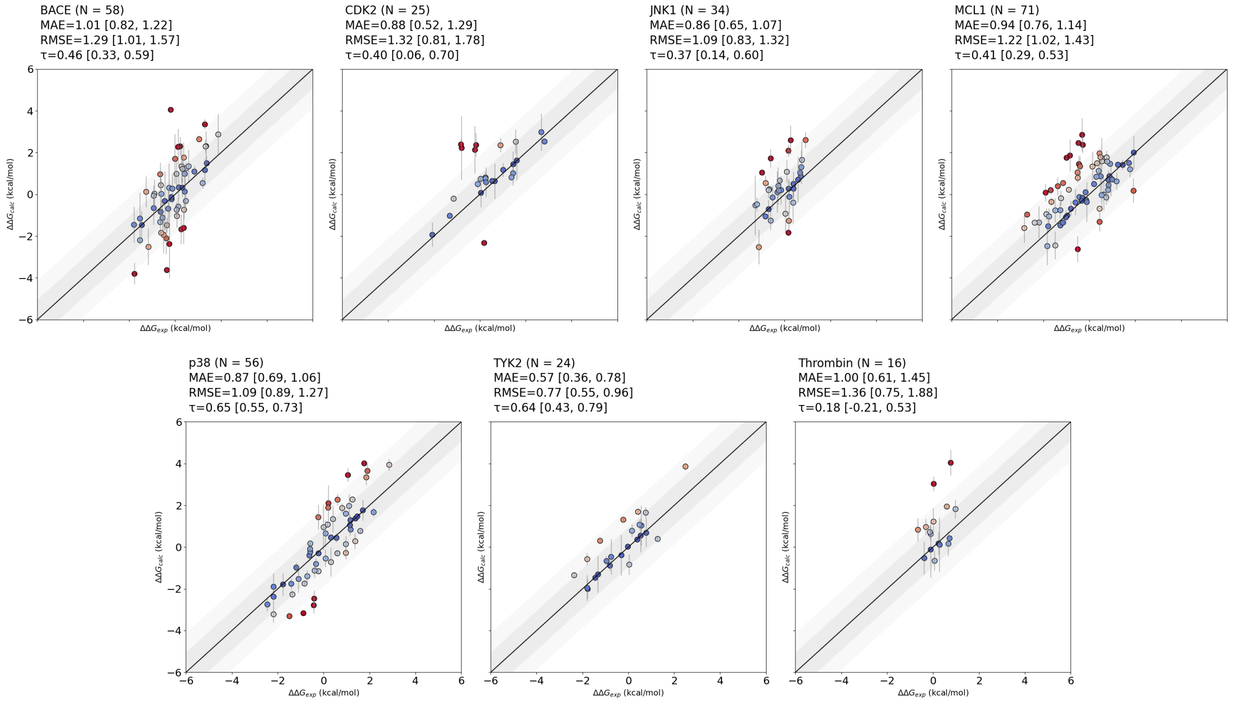

Figure S1: Scatterplots of predicted $\Delta\Delta G$ values for each evaluated system using AceFF 1.0. The grey shaded areas represent absolute error thresholds of 1 kcal/mol and 2 kcal/mol. Additional metrics, including mean absolute error (MAE), root mean square error (RMSE) and Kendall tau correlation (τ) are also displayed. 95% confidence interval values are shown in brackets.

0.4 RESP vs AM1BCC comparison

Table S5: Comparison charge models ΔG

**Target**	**RESP**			**AM1BCC**			$N_{\textbf{lig}}$
	RMSE	MAE	Kendall (τ)	RMSE	MAE	Kendall (τ)	
BACE	$1.07_{0.85}^{1.27}$	$0.87_{0.68}^{1.09}$	$0.33_{0.07}^{0.57}$	$1.16_{0.81}^{1.50}$	$0.83_{0.59}^{1.11}$	$0.37_{0.12}^{0.59}$	36
CDK2	$1.03_{0.62}^{1.37}$	$0.79_{0.51}^{1.11}$	$0.45_{0.11}^{0.84}$	$1.01_{0.59}^{1.46}$	$0.76_{0.49}^{1.17}$	$0.63_{0.34}^{0.87}$	16
JNK1	$1.03_{0.76}^{1.28}$	$0.83_{0.60}^{1.12}$	$0.54_{0.29}^{0.74}$	$1.10_{0.84}^{1.34}$	$0.90_{0.62}^{1.17}$	$0.54_{0.32}^{0.74}$	21
MCL1	$1.64_{1.31}^{1.95}$	$1.32_{1.04}^{1.65}$	$0.44_{0.27}^{0.60}$	$2.13_{1.73}^{2.51}$	$1.69_{1.28}^{2.13}$	$0.47_{0.29}^{0.62}$	42
P38	$0.99_{0.70}^{1.30}$	$0.75_{0.55}^{0.97}$	$0.64_{0.46}^{0.79}$	$1.03_{0.71}^{1.33}$	$0.78_{0.56}^{1.01}$	$0.49_{0.28}^{0.67}$	34
PTP1B	$1.34_{0.79}^{1.83}$	$0.99_{0.67}^{1.40}$	$0.30_{0.03}^{0.54}$	$1.23_{1.18}^{0.76}$	$0.88_{0.59}^{1.23}$	$0.32_{-0.04}^{0.61}$	23
THROMBIN	$0.82_{0.47}^{1.08}$	$0.62_{0.31}^{0.93}$	$0.55_{0.00}^{0.96}$	$1.62_{1.26}^{1.97}$	$1.45_{1.00}^{1.87}$	$0.75_{0.45}^{1.00}$	11
TYK2	$0.64_{0.44}^{0.84}$	$0.64_{0.43}^{0.84}$	$0.67_{0.45}^{0.86}$	$0.91_{0.66}^{1.13}$	$0.80_{0.59}^{1.00}$	$0.49_{0.08}^{0.77}$	16

Table S6: Comparison of top identifiers between RESP and AM1BCC charge models.

**Target**	**RESP**		**AM1BCC**		$N_{\textbf{lig}}$
	**Top 5 (n/5)**	**FBL Top 30% (%)**	**Top 5 (n/5)**	**FBL Top 30% (%)**	
BACE	1	29	2	47	36
CDK2	3	42	4	40	16
JNK1	4	55	2	27	21
MCL1	2	41	2	41	42
P38	3	47	2	35	34
PTP1B	1	21	3	53	23
THROMBIN	3	72	3	67	11
TYK2	4	29	3	21	16

Table S7: Comparison charge models $\Delta\Delta G$

**Target**	**RESP**			**AM1BCC**			$N_{\mathbf{edges}}$
	RMSE	MAE	Kendall (τ)	RMSE	MAE	Kendall (τ)	
BACE	$1.48^{1.74}_{1.20}$	$1.16^{1.41}_{0.92}$	$0.29^{0.48}_{0.09}$	$1.60^{1.76}_{1.15}$	$1.20^{1.38}_{1.05}$	$0.24^{0.48}_{0.02}$	58
CDK2	$1.50^{1.82}_{1.11}$	$1.21^{1.58}_{0.87}$	$0.20^{0.47}_{-0.07}$	$1.26^{1.56}_{0.95}$	$1.00^{1.27}_{0.69}$	$0.35^{0.57}_{0.14}$	25
JNK1	$1.13^{1.42}_{0.82}$	$0.89^{1.12}_{0.67}$	$0.34^{0.60}_{0.06}$	$1.07^{1.34}_{0.74}$	$0.93^{1.15}_{0.72}$	$0.33^{0.64}_{0.08}$	34
MCL1	$1.91^{2.33}_{1.52}$	$1.43^{1.73}_{1.13}$	$0.42^{0.56}_{0.26}$	$2.61^{2.91}_{2.15}$	$2.00^{2.28}_{1.68}$	$0.27^{0.42}_{0.12}$	71
P38	$1.11^{1.40}_{0.80}$	$0.81^{1.02}_{0.63}$	$0.62^{0.73}_{0.50}$	$1.47^{1.73}_{1.14}$	$1.10^{1.21}_{0.90}$	$0.51^{0.67}_{0.32}$	56
PTP1B	$1.98^{2.64}_{1.24}$	$1.33^{1.83}_{0.94}$	$0.17^{0.40}_{-0.09}$	$1.66^{1.86}_{1.18}$	$1.17^{1.43}_{0.87}$	$0.44^{0.61}_{0.21}$	44
THROMBIN	$1.42^{1.86}_{0.95}$	$1.12^{1.54}_{0.74}$	$0.41^{0.67}_{0.10}$	$1.90^{2.32}_{1.46}$	$1.54^{1.97}_{1.10}$	$0.38^{0.65}_{0.15}$	16
TYK2	$0.88^{1.15}_{0.60}$	$0.66^{0.89}_{0.43}$	$0.53^{0.74}_{0.24}$	$1.32^{1.58}_{1.10}$	$1.10^{1.19}_{0.90}$	$0.37^{0.57}_{0.28}$	24

0.5 Top id comparison

Table S8: Comparison of top molecules identified (Top 5 and Top 30%) across different methods.

**Target**	**GAFF2**		**OPLS4 (FEP+)**		**AceFF 1.0**		$N_{\mathbf{lig}}$
	**Top 5** (n/5)	**Top 30%** (%)	**Top 5** (n/5)	**Top 30%** (%)	**Top 5** (n/5)	**Top 30%** (%)	
BACE	1	29	**3**	**60**	**3**	**60**	36
CDK2	**3**	42	**3**	42	2	**52**	16
JNK1	**4**	**55**	**4**	**76**	3	43	21
MCL1	2	41	**3**	**58**	1	16	42
P38	3	47	2	33	**3**	**50**	34
PTP1B	1	21	**4**	**83**	3	84	23
THROMBIN	3	**72**	**4**	**72**	2	28	11
TYK2	4	29	**5**	**73**	**5**	**73**	16

0.6 1fs vs 2fs

Table S9: Comparison of ΔG values from AceFF RBFE calculations at different timesteps

Target	timestep:1fs			timestep:2fs			$N_{\mathbf{lig}}$
	RMSE	MAE	Kendall (τ)	RMSE	MAE	Kendall (τ)	
BACE	$1.07_{0.83}^{1.37}$	$0.86_{0.66}^{1.09}$	$\mathbf{0.52}_{0.32}^{0.72}$	$\mathbf{0.99}_{0.74}^{1.26}$	$\mathbf{0.79}_{0.61}^{1.00}$	$0.48_{0.27}^{0.67}$	36
CDK2	$\mathbf{0.90}_{0.42}^{1.32}$	$\mathbf{0.68}_{0.37}^{1.00}$	$\mathbf{0.65}_{0.40}^{0.89}$	$0.94_{0.46}^{1.36}$	$0.64_{0.33}^{1.03}$	$\mathbf{0.68}_{0.41}^{0.88}$	16
JNK1	$\mathbf{1.12}_{0.91}^{1.32}$	$0.98_{0.76}^{1.22}$	$0.50_{0.28}^{0.72}$	$1.15_{0.80}^{1.50}$	$\mathbf{0.92}_{0.66}^{1.21}$	$\mathbf{0.55}_{0.35}^{0.73}$	21
P38	$\mathbf{0.77}_{0.55}^{0.97}$	$\mathbf{0.58}_{0.41}^{0.76}$	$\mathbf{0.72}_{0.60}^{0.82}$	$0.89_{0.62}^{1.13}$	$0.67_{0.49}^{0.87}$	$\mathbf{0.72}_{0.60}^{0.82}$	34
THROMBIN	$\mathbf{0.57}_{0.42}^{0.71}$	$\mathbf{0.53}_{0.41}^{0.65}$	$\mathbf{0.44}_{0.02}^{0.82}$	$0.80_{0.47}^{1.08}$	$0.64_{0.37}^{0.94}$	$0.42_{-0.14}^{0.83}$	11
TYK2	$\mathbf{0.47}_{0.23}^{0.66}$	$\mathbf{0.31}_{0.16}^{0.51}$	$\mathbf{0.85}_{0.66}^{0.98}$	$0.74_{0.53}^{0.92}$	$0.59_{0.38}^{0.81}$	$0.80_{0.58}^{0.96}$	16

Table S10: Comparison of $\Delta\Delta G$ values from AceFF RBFE calculations at different timesteps

Target	timestep:1fs			timestep:2fs			$N_{\mathbf{edges}}$
	RMSE	MAE	Kendall (τ)	RMSE	MAE	Kendall (τ)	
BACE	$\mathbf{1.20}_{0.92}^{1.47}$	$\mathbf{0.89}_{0.69}^{1.10}$	$\mathbf{0.49}_{0.35}^{0.62}$	$1.32_{1.05}^{1.62}$	$1.04_{0.83}^{1.27}$	$0.46_{0.31}^{0.59}$	58
CDK2	$1.35_{0.87}^{1.25}$	$0.82_{0.50}^{1.24}$	$\mathbf{0.42}_{0.10}^{0.72}$	$\mathbf{1.32}_{0.85}^{1.17}$	$\mathbf{0.88}_{0.52}^{1.28}$	$0.40_{0.06}^{0.70}$	25
JNK1	$1.22_{0.95}^{1.42}$	$0.98_{0.74}^{1.22}$	$0.22_{-0.05}^{0.47}$	$\mathbf{1.09}_{0.84}^{1.30}$	$\mathbf{0.86}_{0.65}^{1.09}$	$\mathbf{0.37}_{0.14}^{0.59}$	34
P38	$\mathbf{1.04}_{0.85}^{1.20}$	$\mathbf{0.84}_{0.69}^{1.01}$	$\mathbf{0.69}_{0.59}^{0.78}$	$1.10_{0.90}^{1.28}$	$0.88_{0.70}^{1.06}$	$0.65_{0.54}^{0.73}$	56
THROMBIN	$\mathbf{1.07}_{0.74}^{1.36}$	$\mathbf{0.89}_{0.63}^{1.20}$	$\mathbf{0.28}_{-0.07}^{0.58}$	$1.36_{0.75}^{1.88}$	$1.00_{0.57}^{1.47}$	$0.18_{-0.20}^{0.52}$	16
TYK2	$\mathbf{0.55}_{0.36}^{0.73}$	$\mathbf{0.42}_{0.29}^{0.58}$	$\mathbf{0.73}_{0.53}^{0.88}$	$0.77_{0.56}^{0.96}$	$0.57_{0.37}^{0.76}$	$0.64_{0.43}^{0.80}$	24

Table S11: Comparison of top identifiers between AceFF 1fs and 2fs runs

Target	timestep:1fs		timestep:2fs		$N_{\mathbf{lig}}$
	Top 5 (n/5)	FBL Top 30% (%)	Top 5 (n/5)	FBL Top 30% (%)	
BACE	**3**	**75**	**3**	59	36
CDK2	**4**	**54**	3	54	16
JNK1	2	32	**3**	**43**	21
P38	2	47	**3**	**50**	34
THROMBIN	**3**	**56**	**3**	11	11
TYK2	4	81	**5**	**85**	16

0.7 AceFF 1.0 vs ANI-2x

Table S12: Comparison of AceFF and ANI-2x calculations for ΔG values. Only the P38 and TYK2 targets are shown since these are the ones that we could compute all edges in our previous study with ANI-2x

Target	N_{lig}	AceFF 1.0			ANI-2x		
		RMSE	MAE	Kendall (τ)	RMSE	MAE	Kendall (τ)
P38	34	$\mathbf{0.77}_{0.56}^{0.97}$	$\mathbf{0.58}_{0.42}^{0.75}$	$\mathbf{0.72}_{0.60}^{0.81}$	$0.93_{0.75}^{1.11}$	$0.78_{0.61}^{0.96}$	$0.58_{0.41}^{0.73}$
TYK2	16	$\mathbf{0.47}_{0.23}^{0.68}$	$\mathbf{0.31}_{0.15}^{0.49}$	$\mathbf{0.85}_{0.65}^{1.00}$	$0.50_{0.28}^{0.72}$	$0.39_{0.26}^{0.54}$	$0.82_{0.57}^{0.96}$

Table S13: Comparison of AceFF and ANI2x calculations for $\Delta\Delta G$ values. The CDK2 and JNK1 datasets are incomplete due to ANI-2x limitations.

Target	N_{edges}	AceFF 1.0			ANI2x		
		**RMSE**	**MAE**	kendall (τ)	**RMSE**	**MAE**	kendall (τ)
CDK2	22	$1.03_{0.57}^{1.41}$	$0.67_{0.35}^{1.03}$	$0.46_{0.34}^{0.59}$	$\mathbf{0.83}_{0.62}^{1.03}$	$\mathbf{0.72}_{0.58}^{0.94}$	$\mathbf{0.62}_{0.34}^{0.81}$
JNK1	27	$\mathbf{0.92}_{0.70}^{1.13}$	$\mathbf{0.74}_{0.56}^{0.94}$	$\mathbf{0.46}_{0.21}^{0.67}$	$0.90_{0.74}^{1.06}$	$0.68_{0.51}^{0.80}$	$0.43_{0.30}^{0.55}$
P38	56	$\mathbf{1.04}_{0.85}^{1.19}$	$\mathbf{0.84}_{0.69}^{1.00}$	$\mathbf{0.69}_{0.59}^{0.78}$	$1.17_{0.94}^{1.37}$	$0.91_{0.72}^{1.10}$	$0.59_{0.48}^{0.70}$
TYK2	24	$\mathbf{0.55}_{0.36}^{0.72}$	$\mathbf{0.42}_{0.28}^{0.57}$	$\mathbf{0.73}_{0.54}^{0.88}$	$0.56_{0.43}^{0.68}$	$0.47_{0.36}^{0.59}$	$0.67_{0.42}^{0.82}$

0.8 Quantumbind-RBFE speed

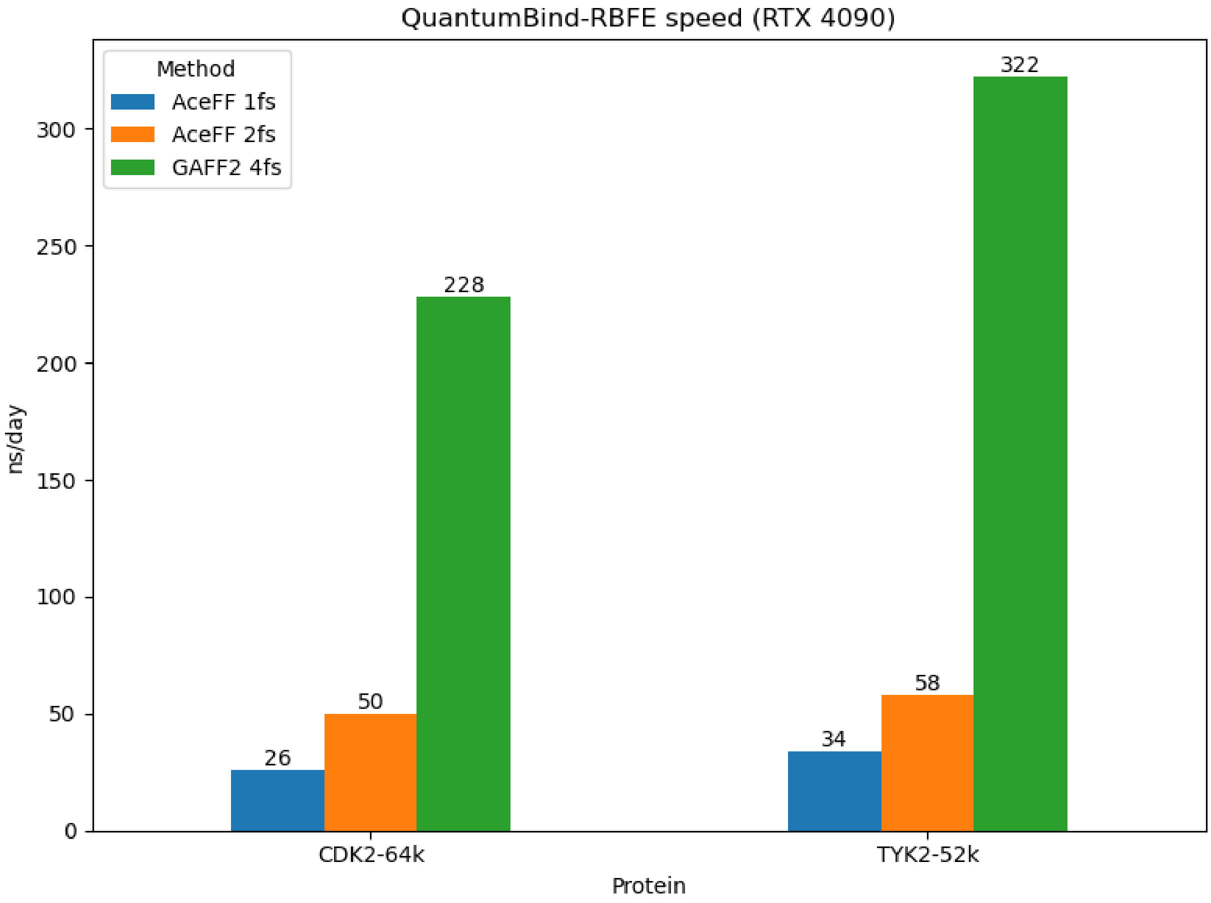

Figure S2: QuantumBind-RBFE simulation speed for various modes. AceFF at 1 and 2fs and GAFF2 at 4fs. All evaluations are done with a RTX 4090 GPU

References

(1) Lu, C.; Wu, C.; Ghoreishi, D.; Chen, W.; Wang, L.; Damm, W.; Ross, G. A.; Dahlgren, M. K.; Russell, E.; Von Bargen, C. D.; others OPLS4: Improving force field accuracy on challenging regimes of chemical space. *Journal of chemical theory and computation* **2021**, *17*, 4291–4300.
